# Furmonertinib demonstrated efficacy in a case of advanced pancreatic cancer with a 19-exon deletion mutation and wild-type KRAS: a case report and literature review

**DOI:** 10.3389/fphar.2026.1761167

**Published:** 2026-07-10

**Authors:** Fengcai Du, Dan Liu, Aihong Jiao, Lulu Xu

**Affiliations:** Departments of Oncology, The Affiliated Yantai Yuhuangding Hospital of Qingdao University, Yantai, Shandong, China

**Keywords:** anlotinib, AST2818, EGFR exon 19 deletion, furmonertinib, pancreatic cancer

## Abstract

In China, advanced pancreatic cancer (PC) is known as the “king of cancers” due to its high treatment difficulty and extremely short survival period. Currently, advanced pancreatic cancer is mainly treated with chemotherapy. The 5-year survival rate is approximately 14.4% for patients with locally advanced pancreatic cancer and only 3% for those with distant metastasis. There are currently no effective targeted drugs for pancreatic cancer. This article reports a case of a patient with advanced pancreatic cancer harboring an EGFR exon 19 deletion. The patient received furmonertinib as third-line therapy, achieving a progression-free survival (PFS) of 6.3 months. Subsequently, drug resistance occurred; increasing the dose of furmonertinib showed no efficacy. Later, a combination of furmonertinib and anlotinib was administered, which successfully overcame the resistance. After follow-up, the patient passed away at home on 22 January 2026. The overall survival time was approximately 38 months. This case suggests that furmonertinib may be effective in patients with pancreatic cancer patients harboring an EGFR exon 19 deletion and that the combination of anlotinib and furmonertinib may overcome resistance to furmonertinib. This is only a case report, and these findings warrant further investigation.

## 1 Introduction

Pancreatic cancer (PC) is a significant public health concern. In 2020, the number of deaths caused by PC was nearly equal to the number of new cases, making it the seventh leading cause of cancer-related deaths in both men and women worldwide (Sung et al., 2021). The survival rate for late-stage diagnosis is significantly lower, with 5-year survival rates of 14.4% for local-stage and 3% for distant-stage disease at the time of diagnosis. The standard treatment for PC involves systemic oncology therapies, such as chemotherapy, targeted/biological therapy, and immunotherapy. The first-line treatment for advanced metastatic PC involves chemotherapy including liposomal irinotecan, oxaliplatin, fluorouracil, gemcitabine, and nab-paclitaxel. The second-line treatment regimen for advanced metastatic PC is selected from regimens not used in the first-line treatment (Salazar et al., 2023). However, the current NCCN guidelines do not recommend any third-line and subsequent treatments for advanced metastatic PC, indicating a lack of effective treatment options, which remains an unsolved clinical challenge. After first- and second-line chemotherapy fail in many patients with advanced pancreatic cancer, no better treatment options are available, leaving them with no choice but to await death. This is an extremely intractable problem faced in current clinical practice.

Furmonertinib (AST2818) is a novel irreversible third-generation epidermal growth factor receptor-tyrosine kinase inhibitor (EGFR-TKI). It has been approved by the National Medical Products Administration (NMPA) of China for the treatment of patients with locally advanced or metastatic non-small cell lung cancer (NSCLC) harboring sensitive EGFR mutations and the T790M resistance mutation (Shi et al., 2021; Shi et al., 2022). Owing to its unique trifluoroethoxypyridine structure, furmonertinib features a broader safety window. Both furmonertinib itself and its major metabolite (AST5902) have been demonstrated to possess high antitumor activity and excellent selectivity (Wu et al., 2022). Previous studies have shown that erlotinib, an EGFR-TKI, is effective in the treatment of pancreatic cancer (Moore et al., 2007). The U.S. Food and Drug Administration (FDA) also approved erlotinib for advanced pancreatic cancer in November 2005.

Then, can furmonertinib exert efficacy in pancreatic cancer? At present, there are no relevant systematic studies. This article reports a case of advanced PC that responded effectively to furmonertinib after progression despite all guideline-recommended regimens, with subsequent combination therapy using anlotinib successfully overcoming drug resistance.

## 2 Case presentation

The study was approved by the Ethics Committee of Yantai Yuhuangding Hospital. Informed consent was obtained from both patients.

## 3 Case report

A 62-year-old female patient was found to have a carbohydrate antigen 19-9 (CA19-9) level of >400 U/ml during a health check-up on 21 November 2022, despite having no symptoms. She visited the Department of Medical Oncology, Yuhuangding Hospital, Yantai City, Shandong Province, China, on 28 November 2022, where a re-examination showed that her carbohydrate CA19-9 level had increased to 981 U/mL. Contrast-enhanced magnetic resonance imaging (MRI) of the abdomen (Figure 1) revealed a space-occupying lesion in the body and tail of the pancreas, presenting as patchy, slightly long T1 and slightly short T2 signals, high signal on diffusion-weighted imaging (DWI), and mild heterogeneous enhancement on contrast scan, with a size of approximately 4.0 × 1.9 cm. The lesion surrounded the splenic vessels, suggesting pancreatic cancer involving the splenic vessels. No cancer metastasis was found on a plain chest computed tomography (CT) scan, and no malignant tumor was detected by colonoscopy. The patient was advised to undergo a pancreatic mass puncture biopsy, but refused, as she intended to seek treatment at a higher-level hospital. On 19 December 2022, the patient visited Shandong Cancer Hospital and underwent “resection of the pancreatic body and tail + total splenectomy + omentectomy + left renal fat capsule resection.” Postoperative pathological results showed: (from resection of the pancreatic body and tail + spleen + omentum) moderately differentiated adenocarcinoma was detected in the pancreas, with lymphovascular tumor thrombus, perineural invasion, invasion of peripancreatic fat, and invasion of the splenic capsule; no cancer was found at the pancreatic stump, spleen, left perirenal fat capsule, or omentum; metastatic adenocarcinoma was present in the transverse mesocolon nodules. Immunohistochemistry showed microsatellite stability, with Claudin 18.2 expression at 1+ (10%) and 2+ (20%). The genetic test results of the resected pancreatic tumor tissue showed that there was a non-mutated deletion mutation in exon 19 of EGFR, a missense mutation in exon 8 of TP53, a 5′exon mutation in exon 7 of CDC73, a frameshift mutation in exon 6 of SMAD4, a missense mutation in exon 2 of MYC, a missense mutation in exon 4 of PDCD1, and the PD-L1 expression TPS was less than 1% with a CPS of 5.

**FIGURE 1:**
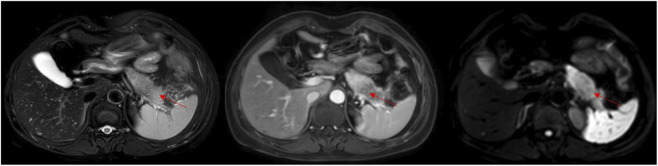
Contrast-enhanced MRI of the entire abdomen at the initial visit on 28 November 2022. The lesion showed slightly long T1 and slightly short T2 signals, a high signal on diffusion-weighted imaging (DWI), and mild heterogeneous enhancement on contrast-enhanced imagining, with a size of approximately 4.0 × 1.9 cm. It surrounded the splenic vessels, suggesting pancreatic cancer (Ca) involving the splenic vessels.

After surgery, the patient received 8 cycles of chemotherapy. The chemotherapy regimen was gemcitabine 1 g on days 1 and 8 + nab-paclitaxel 200 mg on day 1 and 100 mg on day 8. During chemotherapy, the efficacy evaluation indicated stability. CA19-9 decreased from 37.1 U/mL to 29.6 U/mL. Re-examination on 3 April 2023 and 14 July 2023 showed no recurrence.

On 17 October 2023, the re-examination showed that the CA19-9 level was 415 U/mL. On 20 October 2023, re-examination of contrast-enhanced MRI of the abdomen (Figure 2) revealed peritoneal lymph node metastasis, left peritoneal metastasis, abdominal wall metastasis, and metastasis in the abdominal mesenteric region. On 30 October 2023, the patient received local radiotherapy at a dose of 50 Gy/25f. From 15 December 2023 to 28 May 2024, the patient underwent 12 cycles of first-line systemic chemotherapy (oxaliplatin 100 mg on day 1 + irinotecan 230 mg on day 1 + fluorouracil 0.5 g on day 1 + calcium leucovorin 175 mg on day 1 + fluorouracil 3 g via continuous intravenous infusion for 46 h, repeated every 2 weeks). During chemotherapy, the efficacy evaluation indicated stability. The CA19-9 decreased from 591 U/mL to 37.2 U/mL.

**FIGURE 2:**
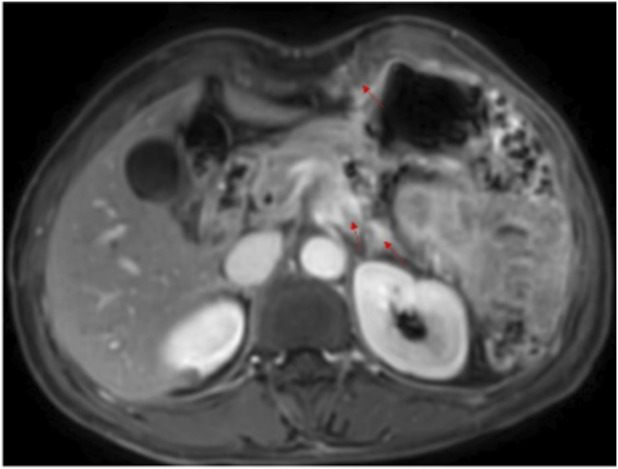
Contrast-enhanced abdominal MRI on 20 October 2023, showing peritoneal lymph node metastasis, left peritoneal metastasis, abdominal wall metastasis, and metastasis in the abdominal mesenteric region.

On 17 June 2024, re-examination showed that the CA19-9 level was 55.6 U/mL. Chest CT showed no evidence of metastasis. Contrast-enhanced MRI of the abdomen revealed postoperative changes in the pancreatic body and tail, spleen, and omentum and significantly enhanced lesions around the retroperitoneal celiac trunk, with an increased extent compared with the previous examination. A consultation with the radiotherapy department was conducted for the retroperitoneal celiac trunk surrounding metastases; re-radiotherapy was not recommended since the lesions around the celiac trunk had previously received radiotherapy. The treatment response was evaluated as progressive disease (PD). From 19 June 2024 to 25 October 2024, the patient received nine cycles of second-line systemic chemotherapy (nimotuzumab 400 mg on day 1 + liposomal irinotecan 86 mg, repeated every 21 days). During chemotherapy, the efficacy evaluation indicated stability. CA19-9 decreased from 55.6 U/mL to 23.9 U/mL.

On 8 November 2024, re-examination showed that the CA19-9 level was 108 U/mL. Chest CT showed no evidence of metastasis. Contrast-enhanced MRI of the abdomen (Figures 3A) revealed that the range of lesions around the retroperitoneal celiac trunk was larger than before; the abnormally enhanced mass at the left-sided abdominal wall incision in the mid-abdomen was also larger than before, which was considered metastatic. The treatment response was evaluated as PD.

**FIGURE 3:**
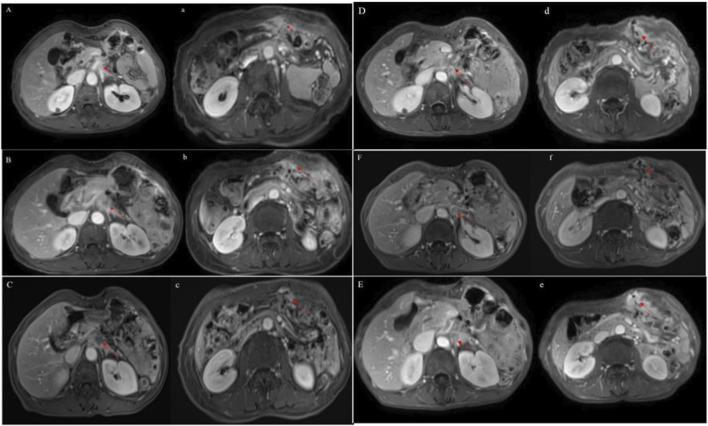
**(A)** Contrast-enhanced MRI of the entire abdomen on 8 November 2024, showing a lesion around the retroperitoneal celiac trunk with a maximum diameter of 2.6 cm. a) Contrast-enhanced MRI of the entire abdomen on 8 November 2024, showing abnormal enhancement at the left-sided abdominal wall incision in the mid-abdomen, which is considered metastatic. **(B)** Contrast-enhanced MRI of the entire abdomen on 10 December 2024, showing a retroperitoneal lesion around the celiac trunk with a maximum diameter of 2.2 cm. b) Contrast-enhanced MRI of the entire abdomen on 10 December 2024, showing no significant change in the abnormally enhanced mass at the left-sided abdominal wall incision in the mid-abdomen compared with the MRI performed on 8 November 2024. **(C)** Contrast-enhanced MRI of the entire abdomen on 21 February 2025, showing a retroperitoneal lesion around the celiac trunk with a maximum diameter of 2.0 cm. c) Contrast-enhanced MRI of the entire abdomen on 21 February 2025, showing a slight reduction in the extent of the abnormally enhanced mass at the left-sided abdominal wall incision in the mid-abdomen compared with the previous examination. **(D)** Contrast-enhanced MRI of the entire abdomen on 19 May 2025, showing a retroperitoneal lesion around the celiac trunk with a maximum diameter of 1.8 cm. d) Contrast-enhanced MRI of the entire abdomen on 19 May 2025, showing a slight increase in the range of the abnormally enhanced mass at the left-sided abdominal wall incision in the mid-abdomen compared with the previous examination. **(E)** Contrast-enhanced MRI of the entire abdomen on 14 July 2025, showing a retroperitoneal lesion around the celiac trunk with a maximum diameter of 1.8 cm. e) Contrast-enhanced MRI of the entire abdomen on 14 July 2025, showing an increase in the extent of the abnormally enhanced mass at the left-sided abdominal wall incision in the mid-abdomen compared with the previous examination. **(F)** Contrast-enhanced MRI of the entire abdomen on 20 August 2025, showing a lesion around the celiac trunk with a maximum diameter of 1.3 cm. f) Contrast-enhanced MRI of the entire abdomen on 20 August 2025, showing a reduction in the metastatic tumor at the left-sided abdominal wall incision in the mid-abdomen.

Oral furmonertinib 80 mg once daily (qd) was initiated on 10 November 2024. On 9 December 2024, re-examination showed that the CA19-9 level was 41 U/mL. Compared with the abdominal MRI on 8 November 2024, the contrast-enhanced MRI of the abdomen (Figures 3B) showed that the retroperitoneal lesion around the celiac trunk was slightly reduced in size: approximately 2.2 × 1.9 cm versus the previous 2.5 × 2.6 cm, with a 15.38% reduction in the maximum tumor diameter. The abnormally enhanced mass at the left-sided abdominal wall incision in the mid-abdomen showed no significant change compared with the previous examination. The treatment response was evaluated as stable disease (SD), and oral furmonertinib 80 mg qd was continued.

On 21 February 2025, re-examination showed that the CA19-9 level was 25.1 U/mL. Compared with the MRI on 9 December 2024, the contrast-enhanced MRI of the abdomen (Figures 3C) revealed that the retroperitoneal lesion around the celiac trunk was slightly reduced in size (2.0 × 1.5 cm vs. 2.2 × 1.9 cm), and the abnormally enhanced mass at the left-sided abdominal wall incision in the mid-abdomen also showed a slight reduction in range. A plain chest CT scan showed no evidence of metastasis. The treatment response was evaluated as stable disease (SD), and oral administration of furmonertinib 80 mg once daily (qd) was continued.

On 19 May 2025, re-examination showed that the CA19-9 level was 49.3 U/mL. Compared with the MRI on 21 February 2025, the contrast-enhanced MRI of the entire abdomen (Figures 3D) revealed that the retroperitoneal lesion around the celiac trunk was slightly reduced in size (1.8 × 1.4 cm vs. 2.0 × 1.5 cm), while the metastatic tumor at the left-sided abdominal wall incision in the mid-abdomen was slightly enlarged. Plain chest CT scan showed no evidence of metastasis. Considering the slow enlargement of the metastatic lesion at the left-sided abdominal wall incision in the mid-abdomen, the dose of furmonertinib was increased to 120 mg once daily.

The patient developed grade III diarrhea outside the hospital, which was partially relieved by oral montmorillonite powder and loperamide (Imodium) for diarrhea treatment. On 14 July 2025, re-examination showed that the CA19-9 level was 66.9 U/mL. The contrast-enhanced MRI of the entire abdomen (Figures 3F) revealed that, compared with the MRI on 19 May 2025, the lesion around the celiac trunk showed no significant change (1.8 × 1.4 cm), while the metastatic tumor at the left-sided abdominal wall incision in the mid-abdomen had enlarged. No evidence of metastasis was found on chest CT. The treatment response was evaluated as PD. The patient received radioactive seed implantation for the metastatic tumor at the left-sided abdominal wall incision in the mid-abdomen on 16 July 2025 and was started on combined treatment with oral anlotinib and furmonertinib on 17 July 2025. The administration of anlotinib was 10 mg orally once daily, for 14 consecutive days, followed by 7 days off, with a 21-day cycle. Furmonertinib was administered as 80 mg orally once daily, continuously without interruption.

The patient had no obvious discomfort outside the hospital. On 20 August 2025, re-examination showed that the CA19-9 level was 118 U/mL. The contrast-enhanced MRI of the entire abdomen (Figures 3E) revealed that, compared with the MRI on 14 July 2025, the lesion around the celiac trunk was reduced in size (1.3 × 1.2 cm), and the metastatic tumor at the left-sided abdominal wall incision in the mid-abdomen was also reduced. No evidence of metastasis was found on chest CT. The treatment response was evaluated as SD. After follow-up, the patient passed away at home on 22 January 2026. The overall survival time was approximately 38 months. The combined treatment with oral anlotinib and furmonertinib was continued. A summary of the treatment course is provided in Table 1. The trend of CA19-9 changes is provided in Figure 4.

**TABLE 1:** Summary of the treatment course.

Date	Event/Treatment	Evaluation/Outcome
2022-12-19	Diagnosed, treated with surgery and 8 cycles of post-operative chemotherapy	2023-10-17 PD
2023-10-30	Received local radiotherapy	-
2023-12-15 to 2024-5-28	12 cycles of first-line systemic chemotherapy	2024-7-17 PD
2024-7-19 to 2024-10-15	9 cycles of second-line systemic chemotherapy	2024-11-8 PD
2024-11-10 to 2025-5-19	Furmonertinib was administered at a dose of 80 mg orally once daily	2025-5-19 PD
2025-05-19	Dose of furmonertinib increased to 120 mg once daily	2025-7-14 PD
2025-07-16	Received radioactive seed implantation	-
2025-07-17	Combined treatment with oral anlotinib and furmonertinib	2025-08-20 SD
2026-1-11	The patient passed away at home	OS 38 m

**FIGURE 4:**
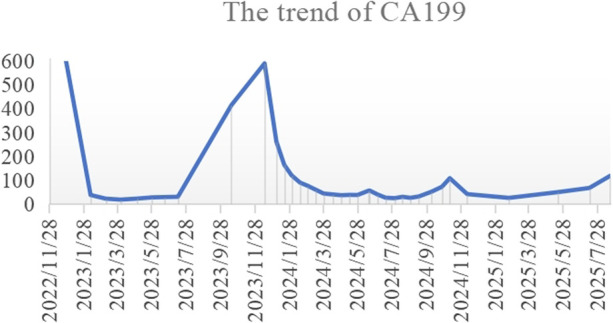
Trend of CA199 changes.

## 4 Discussion

Pancreatic cancer is a highly malignant tumor with a very high degree of malignancy. Its incidence has shown a slow upward trend since 2000. In the United States, it was the fourth leading cause of cancer-related death in 2021 and is projected to become the second leading cause by 2030 (Rahib et al., 2014; Siegel et al., 2021). The latest data released by the American Cancer Society (Siegel et al., 2024) shows that although the 5-year survival rate of pancreatic cancer patients has increased from 3% to 13% compared to 50 years ago, it is still the lowest among all cancers, accounting for nearly 5% of global cancer deaths (Bray et al., 2024). The statistics from the National Cancer Center of the Chinese Academy of Medical Sciences (Wu et al., 2024) show that it is estimated that there will be 76,030 new cases of pancreatic cancer and 68,222 deaths in China in 2024. Pancreatic cancer is undoubtedly a highly lethal malignant tumor. In recent years, with the advancement and widespread application of gene sequencing technology, significant progress has been made in the research of pancreatic cancer, especially in the field of targeted therapy. In the review by Li et al. (2024), detailed explanations were provided regarding the mechanism studies and related clinical trials targeting genes such as KRAS, PARP, PD1/PDL1, SMAD4, RET, and NTRK in the treatment of pancreatic cancer. For example, the preliminary clinical study on the KRAS-G12C inhibitor (CodeBreaK 100) aimed to evaluate the safety and efficacy of sotorasib (AMG510) in various advanced solid tumors with KRAS-G12C mutations (Hong et al., 2020). This study included 12 pancreatic cancer patients, and 1 of them achieved a confirmed objective response after several doses of sotorasib treatment. The other 8 patients maintained stable conditions, with a disease control rate of 75%. The subsequent analysis of the CodeBreak 100 trial showed that sotorasib was extended to 38 patients with metastatic pancreatic ductal adenocarcinoma who had previously received systemic treatment (Strickler et al., 2023). Eight patients (21%) confirmed partial response, and the disease control rate was 84%. The median progression-free survival and overall survival were 4–7 months. However, the case reported in this article involved a patient with wild-type KRAS and an EGFR exon 19 deletion mutation.

EGFR is believed to play a role in the pathogenesis of pancreatic cancer. 90% of pancreatic cancers show high expression of EGFR, and high EGFR expression is usually associated with a poor prognosis. High EGFR expression is mainly caused by EGFR gene amplification or gene mutation. When the EGFR gene is amplified, excessive receptors are expressed on the cell surface, causing uncontrolled cell growth and division, promoting the transformation of normal cells into cancer cells, and supporting the continuous survival of cancer cells. The German II-stage study preliminarily confirmed that patients with high EGFR expression benefited significantly from nintedanib treatment. High EGFR expression is expected to become a new advantageous population for nintedanib. NOTABLE is a prospective, randomized controlled, double-blind, multicenter III-stage clinical study conducted in China, comparing the efficacy of nintedanib combined with gemcitabine with placebo combined with gemcitabine in the treatment of KRAS wild-type locally advanced or metastatic pancreatic cancer. The results indicated that compared with placebo combined with gemcitabine, nintedanib combined with gemcitabine significantly prolonged overall survival, with an mPFS of 4.2 months versus 3.6 months and a median mOS of 10.9 months versus 8.5 months. EGFR mutations alter receptor behavior, resulting in constitutive receptor activation that promotes abnormal cell growth. EGFR mutations and KRAS mutations are usually mutually exclusive. Therefore, EGFR mutations are rarely observed in patients with pancreatic cancer (Ma et al., 2023). The case reported in this article also involved a patient with wild-type KRAS and an EGFR exon 19 deletion mutation. The patient also benefited from the combination of nimotuzumab and chemotherapy as second-line treatment and from treatment with the EGFR-TKI, furmonertinib, as third-line therapy. This demonstrates the significance of precise targeted therapy in the treatment of advanced pancreatic cancer.

Moore et al. (2007) conducted a phase III trial to investigate the efficacy of the EGFR-TKI erlotinib combined with gemcitabine versus gemcitabine alone in patients with advanced pancreatic cancer. An intent-to-treat analysis demonstrated that overall survival was significantly prolonged in the erlotinib/gemcitabine arm (*p* = 0.038) (Moore et al., 2007). Consequently, the U.S. Food and Drug Administration (FDA) approved erlotinib for the treatment of advanced PC in November 2005. However, critics have questioned whether a hazard ratio (HR) of 0.82 for the primary endpoint of overall survival is sufficient to define the trial as successful (Ko, 2023). Results from the LAP07 Randomized Clinical Trial by Hammel et al. (2016) showed that in patients with locally advanced pancreatic cancer whose disease was controlled after 4 months of induction chemotherapy, there was no significant difference in overall survival between chemoradiotherapy and chemotherapy alone. In other words, the efficacy of EGFR-TKI in pancreatic cancer remains highly controversial.

Furmonertinib is an orally active, mutant-selective EGFR inhibitor. It can inhibit EGFR-active mutations and the T790M acquired resistance mutation. So, as a third-generation EGFR-TKI, can furmonertinib control the proliferation of pancreatic cancer cells by inhibiting EGFR-active mutations? Apart from case reports, there are no in-depth studies on this topic (Ma et al., 2023). The advanced pancreatic cancer patient reported in this article had disease progression after receiving two rounds of standardized chemotherapy. Immunohistochemistry showed microsatellite stability, with Claudin 18.2 expression at 1+ (10%) and 2+ (20%). The genetic test results of the resected pancreatic tumor tissue indicated that there was a non-mutated deletion mutation in exon 19 of EGFR, a missense mutation in exon 8 of TP53, a 5′exon mutation in exon 7 of CDC73, a frameshift mutation in exon 6 of SMAD4, a missense mutation in exon 2 of MYC, a missense mutation in exon 4 of PDCD1, and the PD-L1 expression TPS was less than 1% with a CPS of 5. Therefore, beginning on 10 November 2024, she was treated with oral furmonertinib at a dose of 80 mg per day. The metastatic lesions shrank until the disease progressed on 19 May 2025, with a PFS of 6.3 months. A PFS of 6.3 months for the third-line treatment of advanced pancreatic cancer is a length that has not been achieved by other drugs so far. Moreover, the patient at a dose of 80 mg per day had no intolerable adverse reactions. Research by Shi et al. (2024) indicates that increasing the oral dose of furmonertinib may overcome resistance to third-generation EGFR-TKIs in lung adenocarcinoma (Bray et al., 2024). Then, can dose escalation also overcome resistance in pancreatic cancer? The case reported in this article shows a negative result. On 19 May 2025, the patient developed resistance to 80 mg of oral furmonertinib per day. Increasing the dose to 120 mg per day not only failed to control the disease but also caused a grade III diarrhea adverse reaction. This might be a sign that patients with advanced pancreatic cancer who have an EGFR exon 19 deletion could potentially benefit from drugs such as vemurafenib, which are EGFR-TKIs.

Research presented by Han et al. (2025) demonstrated that the combination of anlotinib and furmonertinib can overcome resistance to third-generation EGFR-TKIs in lung adenocarcinoma. However, is this regimen applicable to pancreatic cancer? In the case reported herein, after the patient developed resistance to furmonertinib, treatment with a combination of anlotinib and furmonertinib (80 mg orally once daily) resulted in tumor shrinkage and effective disease control. Furthermore, the patient’s condition remains stable to date.

This patient underwent radioactive seed implantation therapy for the metastatic tumor at the left abdominal wall incision site in the middle of the abdomen on 16 July 2025. Subsequently, anlotinib was also combined. Therefore, the shrinkage of the patient’s lesions might also be attributed to radioactive particle implantation. However, the stability of the patient’s other lesions and the subsequent PFS should be attributed to the systemic treatment, namely, the combination of furmonertinib and anlotinib. Even so, given that this article is only a single case report and the strength of the evidence is limited, the efficacy of combining anlotinib with furmonertinib for the treatment of pancreatic cancer still requires validation through further clinical studies.

## 5 Conclusion

This case suggests that furmonertinib may be effective in patients with pancreatic cancer harboring an EGFR exon 19 deletion and that the combination of anlotinib and furmonertinib may overcome resistance to furmonertinib. This is only a case report, and these findings warrant further investigation.

## Data Availability

The original contributions presented in the study are included in the article/supplementary material; further inquiries can be directed to the corresponding authors.
